# The complete mitochondrial genome of *Ilyonectria* sp. (Hypocreales: Hypocreomycetidae)

**DOI:** 10.1080/23802359.2018.1536490

**Published:** 2019-07-12

**Authors:** Mei Yang, Qiang Li, Cheng Chen, Wenli Huang, Chengyi Liu

**Affiliations:** a Panzhihua City Academy of Agricultural and Forest Sciences, Panzhihua, Sichuan, China;; b Biotechnology and Nuclear Technology Research Institute, Sichuan Academy of Agricultural Sciences, Chengdu, Sichuan, China;; c Institute of plant protection, Sichuan Academy of Agricultural Sciences, Chengdu, Sichuan, China

**Keywords:** *Ilyonectria*, mitogenome, phylogenetic analysis

## Abstract

In the present study, we presented the complete mitochondrial genome (mitogenome) of *Ilyonectria* sp. The complete mitogenome of *Ilypnectria* sp. was composed of circular DNA molecules, with a total length of 34, 584 bp. The base composition of this mitogenome is as follows: A (35.31%), T (35.92%), G (15.93%), and C (12.83%). The mitogenome contains 18 protein-coding genes, 2 ribosomal RNA genes (rRNA), and 25 transfer RNA (tRNA) genes. The taxonomic status of the *Ilyonectria* sp. mitogenome exhibits a closest relationship with *Ilyonectria destructans*.

The *Ilyonectria radicola* complex is a group of soil fungi that is widely distributed in the world (Farh et al. 2018). Over a dozen species have been described in this complex (Aiello et al. 2017; Cabral et al. 2012a). These fungi were reported to be associated with the symptoms of root rot on a broad range of herbaceous and woody host plants, such as ginseng, azalea, poplar, spruce, and apple, but also in stems or cankers of diseased trees (Cabral et al. 2012b; Chaverri et al. 2011). They can also exist as endophytes in the roots of apparently healthy plants, where they may inhibit other fungal root pathogen and maintain the health of the host (White et al. 1962). The mitogenome of *Ilyonectria* sp. reported here will promote further understanding of the population genetics, evolution, and taxonomy of this fungal complex.

The specimen (*Ilyonectria* sp.) was isolated from healthy roots of apple trees in Chengdu, Sichuan, China (104.50 E; 34.15 N) and was stored in Sichuan Academy of Agricultural Sciences (No. SaIp_3). This strain was identified belonging to the *Ilyonectria radicola* complex. The total genomic DNA of *Ilyonectria* sp. was extracted using a Fungal DNA Kit D3390-00 (Omega Bio-Tek, Norcross, GA, USA) and purified through a Gel Extraction Kit (Omega Bio-Tek, Norcross, GA, USA). Purified genomic DNA was stored in the sequencing company (BGI Tech, Shenzhen, China). Sequencing libraries were constructed with purified DNA following the instructions of NEBNext® Ultra™ II DNA Library Prep Kit (NEB, Beijing, China). Whole genomic sequencing was performed by the Illumina HiSeq 2500 Platform (Illumina, SanDiego, CA). Multiple steps were used for quality control and de novo assembly of the mitogenome according to Qiang et al. (2018). Briefly, the SPAdes 3.9.0 software was used to assemble the mitogenome of *Ilyonectria* sp. (Bankevich et al. 2012). Gaps among contigs were filled using MITObim V1.9 (Hahn et al. 2013). The complete mitogenome was annotated using the MFannot tool (Valach et al. 2014), combined with manual corrections. tRNA genes were predicted using tRNAscan-SE v1.3.1 (Lowe and Chan 2016).

The total length of *Ilyonectria sp.* mitogenome is 34, 584 bp. This mitogenome was submitted to GenBank database under accession No. MF924828. The circular mitogenome contains 18 protein-coding genes, 2 ribosomal RNA genes (*rns* and *rnl*), and 25 transfer RNA (tRNA) genes. The base composition of the genome is as follows: A (35.31%), T (35.92%), G (15.93%), and C (12.83%).

To validate the phylogenetic position of *Ilyonectria* sp., we construct the phylogenetic trees of 13 closely related species based on the nucleotide sequences of the 15 core protein-coding genes (PCGs) (*atp6, atp8, atp9, cob, cox1, cox2, cox3, nad1, nad2, nad3, nad4, nad4L, nad5, nad6*, and *rps3*), in addition to the *rns* and *rnl* mitochondrial genes. Bayesian inference (BI) phylogenetic methods were used to construct phylogenetic trees using the combined gene datasets with MrBayes v3.2.6 (Ronquist et al. 2012). Bayesian posterior probabilities (BPP) were calculated to assess node support. As shown in the phylogenetic tree (Figure 1), the taxonomic status of the *Ilyonectria* sp. based on combined mitochondrial gene dataset exhibits a closest relationship with *Ilyonectria destructans* (Okorski and Majchrzak 2012).

**Figure 1.**
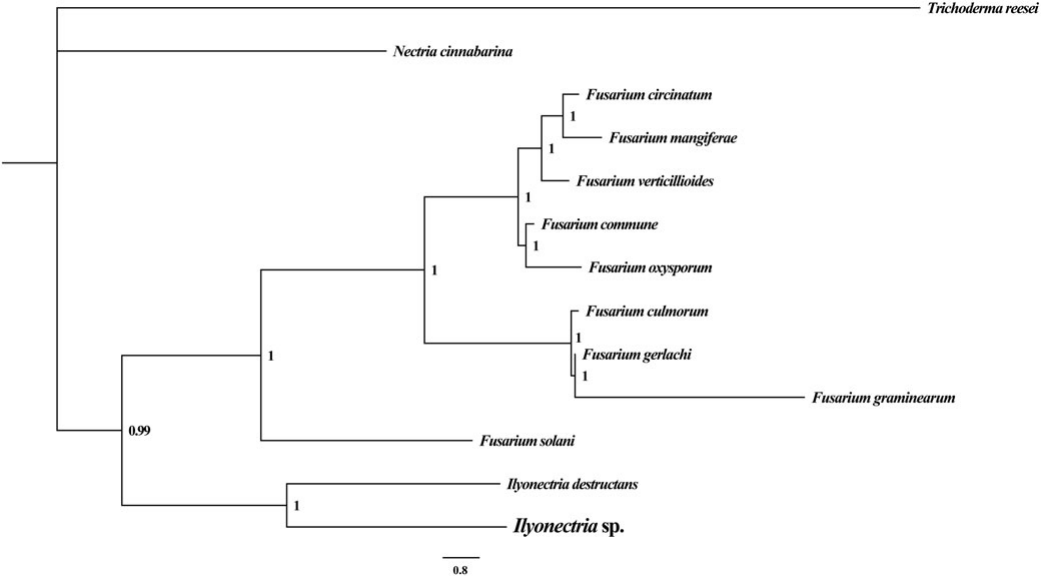
Molecular phylogenies of 13 species based on Bayesian inference analysis of the combined mitochondrial gene set (15 core protein-coding genes + two rRNA genes). Node support values are Bayesian posterior probabilities (BPP). Mitogenome accession numbers used in this phylogeny analysis: *Fusarium circinatum* (NC_022681), *Fusarium commune* (NC_036106), *Fusarium culmorum* (NC_026993), *Fusarium gerlachii* (NC_025928), *Fusarium graminearum* (NC_009493), *Fusarium mangiferae* (NC_029194), *Fusarium oxysporum* (NC_017930), *Fusarium solani* (NC_016680), *Fusarium verticillioides* (NC_016687), *Ilyonectria destructans* (NC_030340), *Nectria cinnabarina* (NC_030252), *Trichoderma reesei* (NC_003388).
